# How Explicit and Implicit Test Instructions in an Implicit Learning Task Affect Performance

**DOI:** 10.1371/journal.pone.0053296

**Published:** 2013-01-09

**Authors:** Arnaud Witt, Ira Puspitawati, Annie Vinter

**Affiliations:** 1 LEAD-CNRS (Laboratoire d'Etude de l'Apprentissage et du Développement – Centre National de la Recherche Scientifique), University of Bourgogne, Dijon, France; 2 University of Gunadarma, Jakarta, Indonesia; Goldsmiths, University of London, United Kingdom

## Abstract

Typically developing children aged 5 to 8 years were exposed to artificial grammar learning. Following an implicit exposure phase, half of the participants received neutral instructions at test while the other half received instructions making a direct, explicit reference to the training phase. We first aimed to assess whether implicit learning operated in the two test conditions. We then evaluated the differential impact of age on learning performances as a function of test instructions. The results showed that performance did not vary as a function of age in the implicit instructions condition, while age effects emerged when explicit instructions were employed at test. However, performance was affected differently by age and the instructions given at test, depending on whether the implicit learning of short or long units was assessed. These results suggest that the claim that the implicit learning process is independent of age needs to be revised.

## Introduction

Implicit learning covers all forms of learning that operate without individuals intentionally deciding to learn or being aware that they are modifying their long-term behavior. This type of learning plays a role in the acquisition of various abilities, such as native [1], [2] and second-language learning [3], the acquisition of sensory-motor behaviors [4], social rules [5], or reading [6] and writing activities [7], [8].

Most of the studies conducted within a developmental perspective have suggested that implicit learning capacities do not develop with age, thus providing support for the claim made by Reber [5]. Roter [9], cited by Reber [5], observed invariant learning performance on an artificial grammar paradigm in 6 to 7, 9 to 11 and 12 to 15-year-old children. In this paradigm, participants are usually exposed to a subset of grammatical strings generated by a finite-state grammar in which, for instance, the strings can be composed of printed consonants. The grammar defines the transition rules between events. Participants are then tested to see whether they can discriminate between new grammatical and nongrammatical strings. The results show that participants recognize grammatical strings at a significantly above-chance level, as if they had discovered the rules of the grammar. These findings were subsequently confirmed by Fischer [10] in 9 to 11-year-olds and by López-Ramón [11] in children aged from 7 to 12 years. The robustness of these results has also been demonstrated in serial reaction time tasks, another classical paradigm used to study implicit learning [12]. In this paradigm, the participants are asked to react as quickly as possible to the appearance of stimuli by pressing keys corresponding to the locations of the targets on the screen. Without them knowing it, the participants are shown a repeated sequence of target locations interspersed by a number of random trials. The results show that reaction times improve on repeated compared to random sequences. Meulemans, Van der Linden and Perruchet [13] concluded that children aged 6–7 and 10–11 years and adults all exhibit the same implicit learning capacities in this type of task. Moreover, in a study conducted by Thomas and Nelson [14], the learning performances of 4 to 10-year-old children were also found to be invariant. Other studies, conducted using a paradigm in which new graphomotor behaviors were implicitly induced in children, have revealed invariant implicit learning capacities in children between 4 and 10 years of age [15]–[17]. Furthermore, Litke [18] and Karatekin, and Marcus and White [19] contrasted two types of learning conditions, an incidental implicit learning condition and an intentional explicit condition, and found that implicit learning performances were globally independent of age, whereas explicit learning performances significantly improved with age. Finally, support for age-independent implicit learning processes has also been provided by studies involving elderly people showing that young and old adults exhibit similar implicit learning capacities (e.g. [20], [21]).

Interestingly, a small number of conflicting results concerning the relation between age and implicit learning have also been reported in the literature. Maybery, Taylor and O'Brien-Malone [22] observed increasing learning performance in an incidental covariation task between the ages of 6 and 12 years, with younger children predicting the location of a target less well after training than the oldest ones. Reporting their incidental learning task, Karatekin et al. [19] identified a number of developmental changes that suggested that the youngest children in their study were more sensitive to interference effects between the blocks presented during the task and took longer to learn the sequences at the start of the task than the older children. The authors considered that these findings were related to similar effects observed in the elderly [23], [24]. A decline in implicit learning capacities in old people has been reported in an experiment in which structurally complex material was presented for processing during the training phase [25], as well as in a study in which a dual task was used during training instead of a single task [26]. Recently, Arciuli and Simpson [27] found age effects in a visual statistical learning task carried out in children between 5 and 12 years of age and showed that they occurred regardless of the durations of presentation of the stimuli. Developmental influences were also reported in a study comparing artificial grammar learning in typically developing children and children with developmental dyslexia [28].

Different hypotheses have been suggested to account for these age effects. Age-related differences in implicit learning performance are expected to emerge when complex material is presented, when conceptual components have to be processed, or when the underlying knowledge required by the task itself develops with age (e.g. [23], [29], [30]). Another, as yet untested, hypothesis, suggests that age effects could be due to the intrusion of explicit influences during the task, and particularly during the task performed at test [15], [31]. Indeed, in most cases, the unconscious influences that are thought to have an effect during implicit training are evaluated at test on the basis of behavioral performance on which the participants explicitly focus. The present study attempts to demonstrate that the probability of age effects being observed in implicit learning may be dependent on the possible intrusion of explicit influences during the task proposed at test. The instructions given at test were manipulated in such a way that they either did or did not make explicit reference to the training phase. One point needs to be made in order to prevent any misunderstanding relating to the use of the term “explicit”. In the experiment presented here, the participants experienced the same implicit training phase. The two groups of participants differed only in the instructions received at test, following the same period of implicit training. Consequently, we did not contrast an implicit and an explicit learning condition, but two implicit learning conditions which were followed by either implicit or explicit instructions at test. We employed an artificial grammar paradigm, which has been rarely used in children, followed by a generation test performed in response to either implicit or explicit instructions. The artificial grammar was structured using colors and made it possible to generate “grammatically correct” colored flags of different lengths. The innovative method employed here had already been used in two recent studies which have reported efficient implicit learning capacities in children aged between 5 and 8 years [32], [33].

To limit the potential influence of explicit processes during the task performed at test time, it is important to avoid explicitly drawing the participants' attention to the properties of the material presented during training. However, this is exactly what seems to happen in most studies of artificial grammar learning in which the participants are asked to judge, when performing the test, whether or not a new item is grammatical (i.e. similar to the items seen during training). We therefore decided to compare the effects of two types of test instructions: in the implicit instructions, the generation test did not make any mention of the material seen during training, while, in the explicit instructions, the participants were required to generate items that they had seen during training. A generation test with implicit instructions does not require children to make any intentional effort to retrieve information, unlike a generation test presented with explicit instructions [34]. We have to make it clear that the implicit test condition was designed to minimize conscious influences by complying with the Neutral Parameter Procedure (NPP, [15]). Although, to our knowledge, the NPP is the best way of achieving this objective, we cannot be sure that it completely prevented conscious influences.

We expected the participants to perform above chance at test, regardless of the instructions, thereby confirming that our artificial grammar learning task was efficient. Given that performance in this paradigm is probably underpinned by fragment-based learning, especially in the case of the most frequent bigrams or trigrams (e.g. [35]–[38]), implicit learning was measured through the production of grammatical bigrams and trigrams as is usually the case. However, we expected to observe a higher level of implicit learning for the bigrams than for the trigrams given that some authors have reported less efficient learning of more complex units (e.g. [39]). More importantly, we expected age effects to emerge when the test instructions made a direct, explicit reference to the training phase. By contrast, age-invariant learning performance should be observed in a generation test with implicit instructions.

## Methods

### Participants

Fifty-six kindergarten pupils and second graders (27 female and 29 male) aged 5 or 8 years participated in the experiment. The children were divided into two age groups (N = 28 per age group). Within each age group, the participants were randomly assigned either to implicit test instructions (N = 14) or to explicit test instructions (N = 14). Table 1 presents the distribution of the participants to these various conditions.

**Table 1:** Characteristics of the groups (F = female, M = male).

Age Groups	Mean Age (years months)	Sex (F – M)	Test instruction	Number	Mean Age (years, months)	Sex (F – M)
5 years	5,1 (span: 4,9–5,5)	14 – 14	Implicit	14	5	7 – 7
			Explicit	14	5.1	7 – 7
8 years	8,2 (span: 7,9–8,5)	13 – 15	Implicit	14	8,2	6 – 8
			Explicit	14	8,2	7 – 7

None of the children was educationally advanced or retarded and none suffered from attentional or intellectual deficits. Their vision was normal or corrected to normal and their ability to differentiate and name the five colors used in the experiment was briefly tested during a prior verification phase.

### Ethics Statement

This experiment was conducted in accordance with the ethical standards set out in the 1964 Declaration of Helsinki and written parental consent was obtained for each child. The study was approved by an institutional review board which included representatives from the laboratoire d'Etude de l'Apprentissage et du Développement (LEAD), the Centre national de la recherche Scientifique (CNRS) and the Inspection Académique de la Côte-d'Or (Ministry of education), Convention 0482, 2011.

### Material

**Figure 1:**
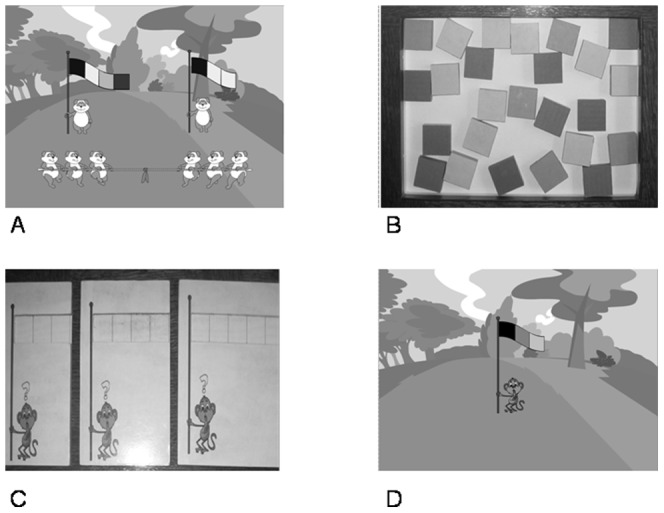
Illustrations of the video game (A. Tug of war tournament; B. Coloured squares; C. Templates; D. Implicit generation phase).

A computer game involving 3-, 4- and 5-color flags was employed, (see Figure 1).

**Figure 2:**
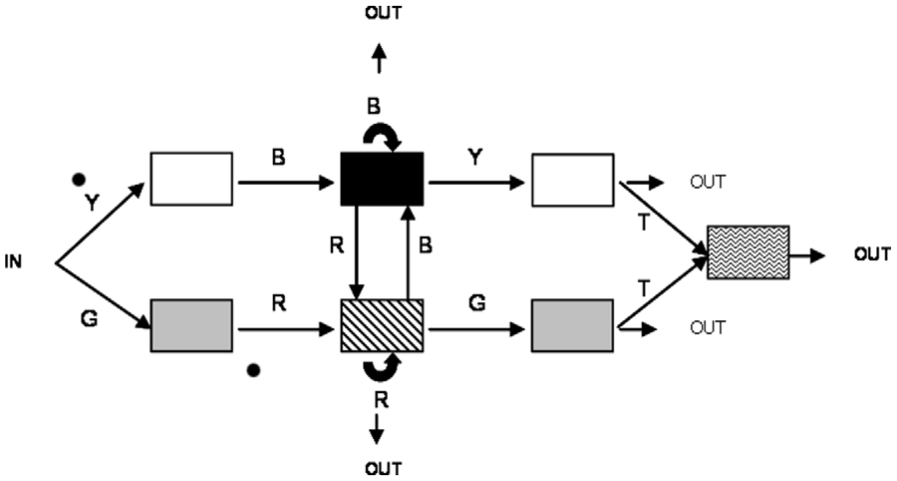
An instanciation of the finite state grammar used in the experiment (the position of the colors was variable).

The flags were produced by a finite state grammar which determined the transitions between five colors (blue, green, red, yellow and turquoise). The grammar made it possible to generate 10 bigrams, 20 trigrams and 42 quadrigrams. The positions of the colors could be switched in the grammar. This resulted in 14 different outcomes and each child saw one of these outcomes regardless of his or her age and test assignment (for an example of the grammar, see Figure 2).

Eight flags were built: two 3-color flags, three 4-color flags and three 5-color flags. The presented flags contained 7 bigrams, 10 trigrams and 8 quadrigrams, as illustrated by the following example: YBB, GRR, YBBR, YBRG, GRRB, YBBYT, GRRBB, GRRBY. This material made it possible to expose the children to all the possible paths through the grammar. In the implicit test (see Procedure), 25 colored squares (5 blue, 5 green, 5 red, 5 yellow and 5 turquoise) (see Figure 1B) were used by the children to build flags based on three templates representing flags of 3, 4 or 5 squares (see Figure 1C).

### Procedure

#### Presentation and training

The experimental session started with a 20-minute phase of exposure to the material and then continued with a 5 to 10-minute test phase. A prerecorded voice delivered the instructions throughout the task. Once the children felt at ease in front of the computer, they were informed that they were going to play a video game. The experimenter stayed next to the children to make sure that their attention remained focused on the screen. They were then told to get ready and the game started playing the following prerecorded instructions: “Hello, today the pandas have organized a “tug-of-war” tournament. Each team of pandas will show you its pretty flag. Press “start” to see the first team's flag”. The colors of the flag were displayed sequentially from left to right for 500 ms each. The complete flag was then displayed for one second and the children heard the instruction “Now, press “start” to see another team's flag”. The 8 flags were seen one at a time in a random order. The instructions then continued: “Now, the tournament is going to start. Press “start” to see the first team's flag” (the flag was displayed, one color at a time, followed by the sound of a trumpet). “Press “start” to see the second team's flag” (the flag was displayed). “Now, press “start” to start the match”. The first team of pandas played tug-of-war against the second (see Figure 1A), with the two flags remaining visible until one of the teams won. The match concluded with a brief animation. This procedure was repeated for 16 matches, so that the children saw the 8 flags 5 times each (once during the presentation phase and 4 times during the matches). All the teams won and lost twice and the position (right or left) of the winning team was random.

#### Test

The training phase was followed by a 5 to 10-minute test phase which started with the prerecorded voice introducing the children to the second part of the game. At this point, one of two different screens was presented depending on the instructions corresponding to the test condition, either implicit or explicit, to which the participants were assigned.

#### Generation test with implicit instructions

In this condition, a monkey holding a blank flag appeared on the screen. The pre-recorded instructions introduced the children to this situation as follows: “The following day, it is the monkeys' turn to play tug of war. Oh, look! The monkey has forgotten to put colors on its flag. You can help him! You know how to make pretty flags, so help the monkey by placing the colors you want on the flag that you see in front of you. Do it now!” Five other monkeys holding blank flags appeared in succession and the children were given the same instructions. The children were asked to produce 3 flags of 3, 4 and 5 colors respectively (random order) by using the 25 colored squares randomly displayed in front of them (see Figure 1B) to fill in a blank pattern consisting of either 3, 4 or 5 empty boxes, depending on the length of the flag. As each flag was completed, the experimenter recorded the corresponding colors using the numeric keypad (see Figure 1D). Each time a flag had been completed, the 25 colored squares were placed in front of the child again. This phase ended when the child pressed the “start” key to watch 3 matches during which the monkeys' flags were displayed. This was followed by an animation congratulating the children before the experimenter registered the data by pressing the “register” button. Throughout the test, no reference to the flags seen during training was made in order to insure that the condition remained implicit as much as possible. We reasonably assumed that designing a pure implicit task was a vain attempt, as claimed by Perruchet [40]. However, our aim was to reduce as much as possible the intervention of intentional retrieval information processes during the test phase. In this view, we applied the NPP [15] which is, to our knowledge, the most appropriate procedure to prevent conscious influences during learning and test. Indeed, the children were asked to produce “pretty flags” and not flags like those seen in the learning phase. They were therefore free to select the colors of their choice and there were no incorrect or correct responses to the task. Nevertheless, it could be considered that the instructions contacted a tacit message referring to children's prior experience during the training phase. We did not assume that the precautions we introduced in our implicit instructions would preclude the possibility that the children might connect the training phase with the test phase. However, it is our view that they seriously limited this possibility.

#### Generation test with explicit instructions

After the implicit training phase (it is important to recall that this phase was the same regardless of the test that followed), the participants assigned to the explicit test condition were asked to build a whole flag that they were sure they had seen during training. The experimenter gave them the 25 colored squares and introduced the task with the following instruction: “A short while ago, you saw the pandas taking part in the tug of war tournament with their flags. I'm going to ask you a question about the pandas' flags, the flags you have seen. Look at this box; it contains the colors used by the pandas. Can you make a whole flag that belonged to the pandas and that you are absolutely sure you saw during the game? Try to remember the pandas' flags, and make a flag when you are absolutely sure you remember a flag you saw during the game.” The children were not told what length of flag they should construct (one child produced a 6-color flag and the additional color was not taken into account in the analyses). The participants were only asked to produce one flag due to the fact that the instructions clearly stressed the need to be confident in the accuracy of the response. Indeed, a pilot experiment had already revealed that 5-year-olds generally refuse to construct a second flag in response to these instructions. This constraint made it impossible to match the number of generated flags in the explicit and implicit conditions, since several different productions could represent the possible expression of incidental influences in the implicit condition, while the degree of confidence required by the explicit instructions precluded multiple productions.

Finally, the experiment ended with a questionnaire phase. The questions dealt with the experimenter's intentions and the children's explicit perception of the properties of the flags: *“Do you know why I asked you to take part in this game? What do you think I observed when you played in this game? Have you noticed something about the colors of the flags? About how the flags were made?”* None of the children who performed the implicit test spontaneously linked the test episode to the training phase. *The children mainly evoked the length of the flags, the help given to the monkeys, or the colors they had seen: “You wanted to see if I can make flags.” “You wanted me to make flags for the monkeys.” “You wanted me to make the pandas and the monkeys play.” “There were some blue, red, green, light blue and yellow colors in the flags.” “There were small, medium-sized and big flags.”* For the children assigned to the explicit condition, they understood the task demands and explicitly linked the training phase to the test episode: “You wanted to see if I could remember the pandas' flags.” “You asked me to make the same flag as those of the pandas.” “You wanted me to use the same colours as the pandas to make a flag”. However, in the implicit as well as in the explicit test condition, none of the answers spontaneously evoked the presence of regularities in the color sequences. These data did not provide any other interesting information and were not analyzed further.

#### Coding of the data

The bigrams and trigrams present in the flags constructed at test by the children were coded as grammatical if they matched the bigrams and trigrams present in the training material. Whereas AGL performance is probably underpinned by fragment-based learning (e.g. [35]–[38]), assessing learning on the basis of bigram and trigram production is not incompatible with rule- or exemplar-based learning, especially when the training items are representative of the grammar as was the case in our study. Indeed, correct fragment generation could also be accounted for by abstract or instance-based learning. We computed the frequencies of grammatical bigrams and trigrams as a function of flag length. This meant, for example, that a grammatical bigram in a 4-color flag scored .33 (1 occurrence out of 3 possible bigrams), while a grammatical trigram scored .50 (1 occurrence out of 2 possible trigrams). The theoretical proportions of grammatical bigrams and trigrams were computed using both the Monte-Carlo method and an analytical approach which produced the same results. The analytical approach computed the precise theoretical probabilities of producing correct bigrams and trigrams in different cases. Depending on the task they were assigned to, the children were presented with 25 colored squares and were then asked either to produce flags of different lengths (3, 4 and 5 colors) or only one flag (of 3, 4 or 5 colors). Consequently, each draw during the generation test reduced the chance of drawing the same color at random. We generated the entire set of 3-, 4- and 5-color flags for the implicit test condition, and the entire set of flags of the length produced by the subject in the explicit condition, using the 25 colored squares in the drawing-without-replacement condition (e.g., for 3-color flags: 25*24*23 possibilities). Finally, the program counted the number of correct bigrams and trigrams in the theoretically generated set. We therefore decided to employ the theoretical proportions obtained using the analytical approach.

We first checked whether the children's production of grammatical bigrams and trigrams was above chance level in the implicit and explicit test conditions, in order to measure implicit learning effects. Student's t-tests were used to compare the observed proportions of bigrams and trigrams with the theoretical ones at 5 and 8 years of age in the two test conditions. Then, for each participant, we computed the ratio between the observed proportions of grammatical bigrams and trigrams and the corresponding theoretical proportions. ANOVAs with Age and Test instructions as between-subjects factors were run on this variable, which directly captures the learning effect (a value equal to 1 means that the proportion of produced grammatical bigrams or trigrams did not depart from chance level).

## Results

We expected to observe implicit learning effects in the form of proportions of grammatical bigrams or trigrams higher than the corresponding theoretical proportions which would result in random production scores. Though we did not predict sub-chance performance explicitly, previous experiments [32] revealed that spontaneous patterns of production, like repetitions avoidance or the use of the whole set of colours at disposal, influenced generation and led to sub-chance performance when implicit learning influences did not operate (like when the children were exposed to random training items, for instance). Because no learning was still a possible outcome in the implicit or in the explicit condition, sub-chance performance could be expected. Therefore, running 2-tailed tests was appropriate. Table 2 presents the results of the Student's t-tests.

**Table 2:** Student's t-test comparing observed proportions of grammatical bigrams and trigrams produced at test to theoretical ones, as a function of Age (5 vs. 8 years) and Test instructions (implicit vs. explicit).

Grammatical units produced at test	Test instructions	Age	Observed proportions	Theoretical proportions	Student's t-test
Bigrams	Implicit	5 years	.447	.358	* t(13) = 2.78, p<.05
		8 years	.489	.358	* t(13) = 2.66, p<.05
	Explicit	5 years	.357	.358	t(13) = −.02, p = .98
		8 years	.613	.358	* t(13) = 4.88, p<.01
Trigrams	Implicit	5 years	.080	.080	t(13) = 0.13, p = .90
		8 years	.121	.080	t(13) = 1.33, p = .20
	Explicit	5 years	.089	.080	t(13) = .19, p = .85
		8 years	.28	.080	* t(13) = 4.28, p<.01

(* indicates significant differences with *p*<.05 or *p*<.01).

As far as bigram production is concerned, when implicit instructions were used at test, the 5- and 8- year-old children both performed significantly above chance, respectively *t*(13) = 2.78, *p*<.05 and *t*(13) = 2.66, *p*<.05. By contrast, in response to explicit test instructions, only the older participants did not perform at chance level, *t*(13) = 4.88, *p*<.01, whereas the younger ones did, *t*<1. A more complex pattern of results was observed on trigram production. The performance of the participants in the implicit instructions condition corresponded to chance irrespective of age, *p_s_*>.15. In the explicit instructions condition, the 5-years-olds' performance remained at chance level, *t*<1, whereas the 8-year-olds' production of grammatical trigrams was significantly above chance, *t*(13) = 4.28, *p*<.01.

**Figure 3:**
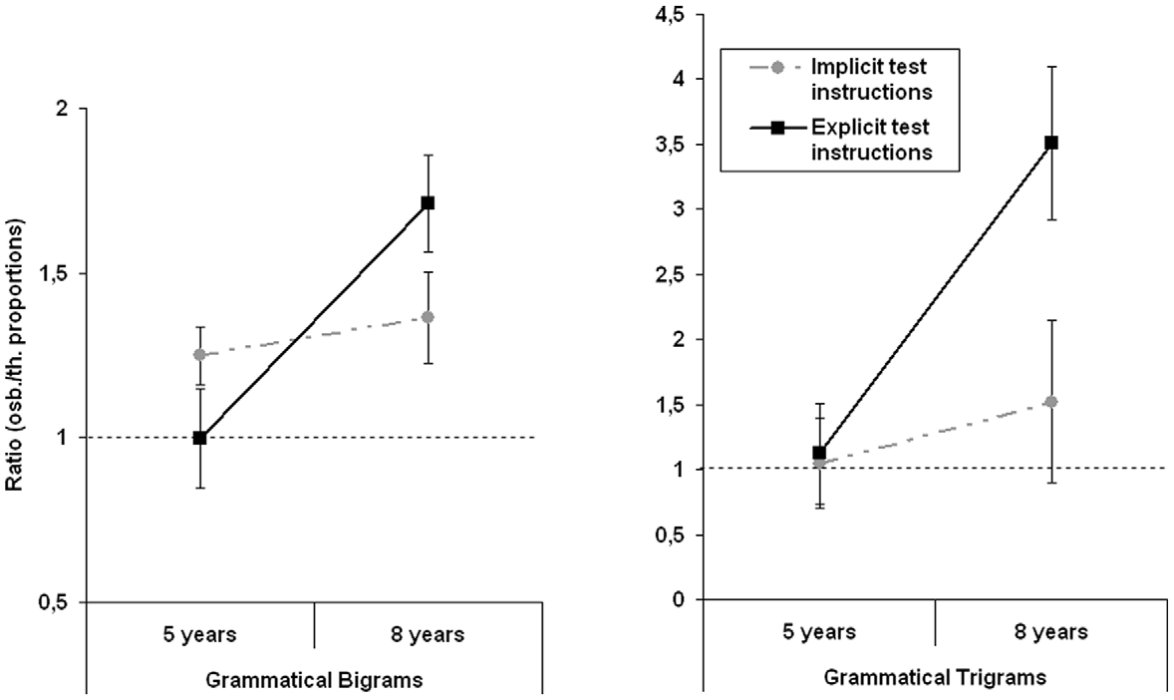
Ratios between observed and theoretical proportions of grammatical bigrams and trigrams as a function of Age (2: 5 years, 8 years) and Test instructions (2: Implicit, Explicit). The error bars correspond to one standard error and the hatched line represents chance level (observed ratio/theoretical ratio = 1).

We then investigated the extent to which the learning performances were affected by age in the two test conditions. To this end, ANOVAs were run on the ratio between observed and theoretical proportions of grammatical bigrams and trigrams, with Test instructions (2: Implicit test instructions vs. Explicit test instructions) and Age (2: 5 years and 8 years) as between-subjects factors. The results are presented in Figure 3.

Concerning the production of grammatical bigrams, the effect of Test instructions did not reach significance, *F*<1, while Age and the Age by Test instructions interaction were both significant, respectively *F*(1, 52) = 9.75, *p*<.01, *η_2;p_* = .16 and *F*(1, 52) = 5.05, *p*<.05, *η_2;p_* = .09. The significant interaction showed that the 8 year-old children performed (*M* = 1.36, *SD* = .51) similarly to the 5-year-olds (*M* = 1.24, *SD* = .33) in the implicit instructions condition, while, following explicit test instructions, the older children (*M* = 1.71, *SD* = .54) outperformed the younger ones (*M* = 1, *SD* = .56). Student's t-tests confirmed that the 5 and the 8 year-old children performed equally under implicit instructions, *t*<1, while the 8 year-olds produced more grammatical bigrams than the younger children under explicit test instructions, *t*(26) = 3.4, *p*<.01. With regard to trigrams, significant Age and Test instructions effects appeared, respectively *F*(1, 52) = 8.15, *p*<.01, *η_2;p_* = .13 and *F*(1, 52) = 4.25, *p*<.05, *η_2;p_* = .075. The Age by Test interaction was just significant, *F*(1, 52) = 3.8, *p* = .05, *η_2;p_* = .07. When the test conditions were analyzed separately, the Age factor failed to reach significance in the implicit instructions condition, *F*<1, while it became significant when explicit instructions were employed at test, *F*(1, 52) = 7.78, *p*<.01, *η_2;p_* = .13, with the 8-year-olds (*M* = 3.51, *SD* = .2.19) outperforming the 5-year-olds (*M* = 1.12, *SD* = 2.34). Student's t-tests corroborated these findings, with the two age groups performing similarly when they received implicit instructions at test, *t*<1, while the older outperformed the younger children in the explicit test instructions condition, *t*(26) = 2.79, *p* = .01.

## Discussion

The aim of this experiment was to test the prediction that age effects in an implicit learning task would emerge when explicit instructions were provided at test. The implicit learning capacities of children aged 5 or 8 years were evaluated using an artificial grammar learning paradigm with a generation test performed in response to implicit or explicit instructions. The results when implicit instructions were given at test revealed that bigrams were learned whatever the age of the participants, whereas no learning effect was observed on the trigrams. In the explicit instructions condition, the older children produced grammatical bigrams and trigrams at above chance level whereas the younger ones did not. Thus, explicit test instructions systematically gave rise to significant age effects, unlike the implicit test instructions. Furthermore, regardless of their age, the children failed to produce the more complex trigrams in response to implicit instruction, whereas the older children succeeded in the explicit instructions condition. The results are discussed with reference to the relationships between implicit learning and age effects, and in relation to the size of the learned units in implicit learning.

### Implicit learning and age effects

Most of the studies run within a developmental perspective have supported Reber's [5] claim that implicit learning capacities do not change with age (e.g. [9], [13], [16], [17], [19]). However, a number of studies have reported conflicting results indicating that performance does indeed develop with age (e.g. [22], [41]). Despite the fact that some authors have suggested that these findings may be due to the unwanted influence of conscious contaminations [15], [31], they nevertheless cast doubt on the robust nature of implicit learning processes. To overcome this problem, Vinter and Perruchet [15] suggested employing the neutral parameter procedure in which implicit learning is assessed on the basis of behavioral components to which the participant's attention has not been drawn. More specifically, the behavior on which the participants have to focus in order to complete the task should differ from the one the experimenter has attempted to induce through the implicit procedure. The present experiment implemented this proposal by comparing two test conditions: one with neutral implicit instructions (compatible with the neutral parameter procedure) and one with instructions that exposed participants to potential explicit influences (incompatible with the neutral parameter procedure). The results showed that in response to implicit instructions, the children's production of grammatical bigrams was the same irrespective of age, with the children performing above chance. By contrast, explicit test instructions resulted in an increase in learning performances between 5 and 8 years of age, with above-chance performances being observed only in the older children. Age interacted significantly with Test instructions. No age effects emerged when the instructions at test were implicit, while they reached significance when explicit instructions were employed.

The results provide support for our hypothesis that age effects are likely to be observed in implicit learning whenever explicit influences intervene at test, that is, whenever the procedure is likely to elicit conscious information retrieval processes. The fact that these processes are less developed in younger children prevents them from accessing the information learned incidentally during the training phase. To use Karmiloff-Smith's concept [42] of representational redescription, these results show that, in our study, the older children performed representational redescription more easily and efficiently than the younger children when the test required them to retrieve information that had not been intentionally encoded.

### About the size of the learned units in implicit learning

Unexpectedly, when implicit learning was assessed through the production of trigrams, learning failure was observed when implicit instructions were employed at test. Indeed, all the participants failed to produce above-chance levels of grammatical trigrams in their flags following implicit test instructions. Interestingly, when explicit instructions were given at test, the older children produced grammatical trigrams at above chance level whereas the younger ones did not. This finding is consistent with our hypothesis that age effects are likely to be observed in implicit learning whenever explicit influences intervene in the test phase. These results suggest that more complex units are less likely to be retrieved implicitly than smaller units by older children and are not retrieved at all by younger children.

Some studies which have used the serial reaction time paradigm have reported complexity effects, with the number of previous locations necessary to predict the next one affecting sequence learning [43], [44]. In some studies using an artificial grammar learning paradigm, the learning of higher-order dependencies has been found to require a longer period of exposure to the material than the learning of first- or second-order dependencies [45], with dependency length-related impairments also being observed [46].

If behavioral adaptation in implicit learning involves attentional processes, as has often been suggested (e.g. [47]–[51]), we might conjecture that the older participants learned more complex units than the younger ones, since the former have greater attentional capacities than the latter [52], [53]. Since the young children failed to produce grammatical trigrams in both test conditions, including in the implicit condition which nevertheless elicited efficient incidental information retrieval processes, our results suggest that the implicit learning of complex units in young children is likely to be constrained by their limited apprehension of the material. In future research, it would be interesting to test whether longer training periods with more repetitions enable young children to learn longer grammatical units. However, this hypothesis does not hold for the older children whose trigram learning performance varied depending on the instructions received at test, despite the fact that the two conditions both followed the same implicit exposure phase. Because only the explicit instructions revealed that the 8-year-olds implicitly learned the grammatical trigrams, it is possible to suggest that explicit information retrieval processes are more efficient than implicit ones when participants are required to access more complex representational units. How can we account for this apparent disadvantage of the implicit retrieval processes? It could be related to the specificities of the generation task when implicit instructions are used. Indeed, an implicit generation task permits the expression of spontaneous patterns of production, such as avoiding repetitions or employing the complete set of available elements, as has been observed among individuals asked to generate random or spontaneous sequences [32], [54]–[57]. Such spontaneous tendencies may enter into conflict with the behaviors that the training task tries to induce through incidental means, i.e. the generation of grammatical flags in our study. Interference resulting from the expression of spontaneous behaviors might be greater when implicit instructions are given at test. Indeed, the guidance provided by explicit instructions orients the retrieval processes and could therefore minimize this interference. Furthermore, we can reasonably argue that small discrete representational units (like bigrams) are less likely to conflict with spontaneous behaviors than longer cognitive units such as trigrams. The first argument is based on the assumption that, throughout the generation task, each color added to the sequence makes the resulting item less representative of the entire set that can be produced with 5 different colors. Indeed, with five colors, 5 monograms, 25 bigrams and 125 trigrams can be generated. There are consequently more candidate events when producing a trigram than a bigram, and there are therefore more concurrent events that are likely to conflict with the implicit influences resulting from the grammatical items seen during training. However, this assumption supposes that the participants selected a bigram or trigram in advance from among a finite set of possibilities in the generation phase and it is far from clear that that is actually the case. Nonetheless, even if the children built their flags step-by-step, interference with spontaneous behavioral tendencies increased as a function of the length of the production. We can indeed consider the following two spontaneous tendencies in the generation of sequences: use of the complete set of available colors or avoiding the repetition of elements [32], [54]–[57]. As the children progressed in the construction of the flag then, if such tendencies were indeed at work, they would have had to be increasingly careful in selecting the colors they had not already used and this would have gradually reduced the scope for the intervention of uncontrolled influences. Explicit instructions, which directed the children's attention to the flags seen during training in the exposure phase, prevented interference from such spontaneous behaviors, thus explaining why the 8-year-old children produced grammatical trigrams in response to explicit but not to implicit instructions.

In conclusion, implicit learning processes appear to be age-independent, provided that the procedure used at test is immune to explicit influences and that the impact of implicit learning is not assessed on the basis of complex representational units. It can be argued that the second constraint imposes a serious limitation on Reber's age-independence postulate. It should be noted that our reasoning can be extended to the IQ-independence postulate also proposed by Reber [5]. Indeed, children with mental retardation are known to exhibit deficits in explicit processes [58]–[62]. Some studies have reported that these children perform as well as typically developing children in implicit learning tasks (e.g. [63]–[67]). However, a number of other studies have revealed that implicit learning or memory performance is correlated with mental age [41], [68]. In the light of the findings reported in this paper, children with mental retardation should perform similarly to typically developing children in an implicit learning task with implicit instructions at test, but not when the instructions are explicit. This prediction could easily be tested in future research.
